# Veterinary Meat-inspection1Read before the Wisconsin State Veterinary Medical Association, February 5, 1896.

**Published:** 1896-03

**Authors:** G. Ed. Leach

**Affiliations:** Milwaukee, Wisconsin


### VETERINARY MEAT-INSPECTION.[1]

By G. ED. LEACH, M.D.C.,
MILWAUKEE, WISCONSIN.

THE subject of veterinary meat-inspection by the State and the necessity for it is one of the great questions that to-day interests alike the breeder, consumer, veterinarian and M. D., and also one which should be presented with the determination to give to the people the assurance of healthful food of this kind, and at the same time they should manifest the most intense interest in and exert every effort to prevent the public from being imposed upon by unscrupulous dealers, both wholesale and retail.

We have in the State of Wisconsin, according to the last census, nearly two millions of people that are consumers of meats in some form or other, from one to three times a day, and the majority have no safeguard thrown around them that what they eat is free from disease. True it is that some are eating inspected meats, but they are in a very small minority compared with those who are obliged to take whatever the dealer nearest their homes has a mind to deal out to them, and a great many are content to have what is considered refuse from the stockyards and wholesale slaughtering-houses. I have seen people of the poorer class and of foreign birth carrying from our stockyards carcasses of animals that had died *in transitu* (and who knows from what cause?), or of premature birth, for the purpose of using them for food. These people are entirely ignorant of the fact that the worst forms of disease are found in such animals.

Many inconsiderate and unscrupulous butchers stand ready to buy anything, either at the yards or in the country, that may be sick and unthrifty, or pregnant in its advanced stage, and, as long as there is no one whose business it is to prevent them from doing so, place it on the market. To the general public they look as well as any, while in these carcasses lurk diseases that may destroy whole families. It is no uncommon thing to pick up the paper and read some startling article of lives lost, and the public simply say, "That was terrible," and go right

[1] Read before the Wisconsin State Veterinary Medical Association, February 5, 1896.

along allowing the same things to continue without entering an objection.

There are to-day in the city of Milwaukee eight wholesalers and about sixty-five jobbers selling meat to retailers, and some retailing themselves. Of these five have government inspection, namely: Swift, Armour, Cudahy, Layton, and Plankinton. The remainder have practically no inspection except that furnished by the Health Department, and generally these have been persons with a pull, who cared more for the welfare of a few friends and the party than the welfare of the public; but, thanks to the civil-service system, this will now be stopped. Even were these inspectors the most capable, the force of help is inadequate to handle with any degree of satisfaction the work forced upon them.

The better class of the people demand and receive the inspected meat by the payment of a higher price for it; but these are only a few compared with the vast number of people who have no protection whatever save that which is furnished by the Health Department police, with no method or rule as to what shall be condemned and what allowed to pass and be consumed. Therefore, the inspectors or police are left to rely wholly upon their own judgment, and in a great many instances they have a price fixed by which their judgment fails to detect anything wrong. The methods employed are wholly inadequate to meet the necessity, and we are worse off to-day than the Jews, or children of Israel, who from their earliest history have demanded and still continue to do so, as a divine law handed down to them from the prophets, that all meats designed for food or sacrifice should be without blemish or disease or pregnancy (four weeks before and two weeks after), and all meats must be examined by a schächter and pronounced by him clean (kosher). Not even the blood can be left in the carcass, and the veins and arteries must be inflated to drain them, as the blood is considered unclean and will pollute them.

Secretary Morton admits that the present system of Government inspection is doing an injustice to the people of the country. You will pardon me for quoting from an article on this subject from a Washington correspondent, dated Nov. 5, 1895: "Secretary Morton will complete his annual report next week. The Secretary will take up the subject of Government inspection of meats, and will point out some of the defects in it as it now exists. The fact that the system fails to protect American consumers while it guards the health of the foreign purchaser of our beef has often been pointed out. The law permits the Federal authorities to condemn, but not to destroy, and this stands in the way of an effectual interference on the part of the Government officials to prevent the consumption of diseased meats in the country. Mr. Morton admits the imperfection of the law and says it is due to our system of government, which leaves such matters largely to the State. He says, however, that there is a remedy for the defect, which is to be found in appealing to the owners of diseased stock or in co-operation with the State Government, and he urges that steps be taken looking to the extension of the National Government's prerogative in this direction." (This refers to the custom of taking animals condemned as diseased to some other place and having them killed.)

Gentlemen, it is my opinion that a uniform plan of meat-inspection, affecting each State as well as the Government, and benefiting the home consumer as well as the foreign, will surely bring beneficial results if carried out. But will it be done?

The National Live-stock Board in session at Chicago, in November, condemned the present system as discriminating against the general good of the people. (See circular from Bureau of Animal Industry, with the last session, date of December 13, 1895.) If this is carried out by the Government inspector then there still remains the obstruction which stands in the way of the inspector. He has no power to destroy, and the owner may take his diseased animals to some local butcher and dispose of the same, if he sees fit to do so. And do you think, gentlemen, that the owners of such stock will have said stock destroyed and thrown in the tanks of their own free will or for the benefit of the public health? Never, until the people rise up in their indignation and demand this to be done through State legislation.

You can see readily that the result of limiting the inspection of meats to that exported out of the State is that the inhabitants are to a great extent obliged to eat the meat that is not good enough for outsiders. The tendency to discriminate in the matter of meat-inspection cannot fail to be harmful to the home consumer. It certainly seems that it is of equally as much importance to have the interests of the consumer of meats at home as carefully guarded as those abroad, and doubtless the best and only method of bringing this about is to secure a uniform standard of inspection.

There is but one way to bring about this uniformity, and that is by a concerted action on the part of the different States and the Government. The Federal law gives the inspectors the right to act in conjunction with the State authorities on all cases *in transitu*, where the State expresses a desire to do so. But what is generally their attitude in such cases? They usually manifest a disposition to rush them off their hands as quickly as possible, with the excuse that they cannot be bothered with such cases, and that they make too much trouble for those in charge of these affairs, for the State and Government inspector is ignored.

There is a section in the Federal law (6-41) which provides for the unloading and feeding of all stock *in transitu*, and a fine of from $100 to $500 for failure; and in case of sickness shall not be reshipped; but with a few exceptions, how many of these unloading points are guarded by either Government or State authorities? In some instances the Health Department take these cases in their hands, and, not having the power to condemn, they are generally censured for making too much trouble and expense for the State. The State code has ample power provided in it for the foundation on which could be built a great work, through which the public might receive some protection if it were enforced, but herein lies the trouble. In looking over the code I find the following acts now in force:

Chapter 187, Sec. 4599, year '89, entitled an act to regulate the selling of unwholesome provisions. Any person who shall knowingly sell any kind of diseased, corrupted, or unwholesome provision, whether for meat or drink, without making the same fully known to the buyer, shall be punished by imprisonment not to exceed six months or by fine not exceeding $100.

Sec. 4605, Chapters 187-189. An act to control diseased animals from running at large. Any person who shall suffer to run at large or shall keep in any place where others animals can have access to or become inflicted by them, any horse, mare, ass, ox, bull, cow, sheep, or other domestic animals owned by him, or in the care or possession, or known by him to be affected by glanders, farcy, or other contagious or infectious disease, or who shall have in his possession in this State any diseased animals, shall be punished by imprisonment in the county jail not more than one year or by fine not to exceed $200.

Sec. 4608, Chapter 187-89. An act regulating the orders of boards of health. Any person who shall wilfully violate any law relating to the public health, or any order or regulation of any board of health, lawfully made and duly published, shall be punished by imprisonment in the county jail not more than three months or by a fine not to exceed $100. Upon complaint made in writing under oath before any magistrate or justice of the peace, charging the commission of any offense against the provisions of any act in his county, it shall be the duty of the district attorney to prosecute the offender.

The general law of the State under Chapter 1492 A has nearly the same construction of the law to govern the State Veterinarian, yet it gives more power to the executor.

I have fully shown by these references that there is now a sufficient legal foundation for the upbuilding of a great work in this line, which, although not adequate, can by being amended from time to time be made to conform to the necessary requirements of this branch of work; but the vital question which has been overlooked is, Who will enforce and carry out the requirements for which there has been no provision? It is left for everybody to look after, and what is everybody's business is no one's business.

It has been conceded by the law that the foundation of the complaints is disease, and who is there to-day able to cope with this stupendous question but the educated and qualified veterinarians? The day has passed when the usefulness of the veterinarian is confined to a dirty, greasy castrating outfit or a few bottles of aconite, belladonna, spirits of nitre, etc. He must be able to cope with these questions of vital importance with the ablest members of the medical profession. And this means a great deal of work in the matter of investigation and research, and this fact becomes more apparent every day, coupled with the necessity of a more concerted action on the part of every member of the profession in order to bring the profession up to the high standard it should possess. Some action should be taken whereby the people shall be profited and the credit placed where it justly belongs.

The first thing that should be considered in this connection to bring about the results to which we should attain is the formation or creation of a board of live-stock commissioners composed of five persons, namely: Two stock-raisers, who shall have a thorough knowledge of feeding, breeding, and sanitary conditions; two veterinarians, who are graduates from reputable colleges and of superior learning, personal skill, and of good moral character; and the fifth a member of the State Board of Health. This shall constitute the Board, who shall, with the State Veterinarians, investigate all cases reported to them of a contagious or infectious nature, and they shall have the power to inspect all herds and, in case of disease, to condemn. There should be a qualified veterinarian on the State Board of Health and on every city board, and in such counties as have no city board of health there should be a county veterinarian who should work with the county board for the purpose of investigating all cases of contagious and infectious diseases reported in the county, and should report such cases to the board of live-stock commissioners. With this plan of work throughout the different States, and they working conjointly with the Bureau of Animal Industry, it would be possible to eradicate all cases of a contagious or infectious nature that might exist, expeditiously and systematically.

Finally, if you guard the food of the people you guard the health, for whatever be the disease, promoting surroundings, a healthy body nourished by healthy food can better withstand the encroachments of disease than the body whose vitality is impoverished by unhealthful food.

